# Use of the adult social care outcomes toolkit (ASCOT) in research studies: an international scoping review

**DOI:** 10.1007/s11136-025-03958-3

**Published:** 2025-04-18

**Authors:** Stacey Rand, Nick Smith, Elizabeth Welch, Stephen Allan, James Caiels, Ann-Marie Towers

**Affiliations:** 1 Personal Social Services Research Unit (PSSRU), University of Kent, Canterbury, UK; 2 Centre for Health Services Studies (CHSS), University of Kent, Canterbury, UK; 3 Health and Social Care Workforce Research Unit (HSCWU), Kings College London, London, UK

**Keywords:** ASCOT, Social care, Long-term care, Quality of life, Outcomes

## Abstract

**Purpose:**

Since the launch of Adult Social Care Outcomes Toolkit (ASCOT) in 2012, there has been increasing interest in use of ASCOT measures in social care research and evaluation, internationally. This scoping review seeks to understand ASCOT use and the methodologies within which the measures have been applied.

**Methods:**

An international scoping review of studies published between January 2012 and July 2024 that utilized ASCOT, excluding measure development and psychometric studies.

**Results:**

Fifty-five articles (11 protocols) reported use of ASCOT. Most reported cross-sectional studies (n = 19) or randomized controlled trials (n = 15) that explored the effectiveness of policy, interventions or systems. ASCOT measures were also applied in mixed methods and other study designs, including qualitative studies. A few studies applied ASCOT to develop theory or conceptual frameworks that relate to care, including how to understand unmet need.

**Conclusion:**

ASCOT measures have been applied, internationally, in a range of ways, with a focus on evaluation studies. Further research is required to explore how ASCOT is used in practice, including care planning. Focus is also needed on ensuring users select the appropriate measure for their study, and widen awareness of adapted versions to support data collection, like ASCOT easy read (ASCOT-ER).

**Supplementary Information:**

The online version contains supplementary material available at 10.1007/s11136-025-03958-3.

## Introduction

Informed by Sen’s capability approach [1, 2], the Adult Social Care Outcomes Toolkit (ASCOT) is a family of instruments that measure *social care-related quality of life* (SCRQoL) [3, 4]. SCRQoL refers to aspects of quality of life (QoL) that are valued and important to people accessing adult social care services and are affected by these services. Adult social care services, also known as long term care (LTC), include a variety of residential and community care services (e.g., homecare, day centres, residential care homes), for adults of all ages. ASCOT was originally developed for LTC economic evaluation, and has been widely-recognised as suitable for this purpose [5–7].

The full suite of ASCOT is summarized in Table 1. To briefly outline the chronology of its development, the preference-weighted self-completion (**ASCOT-SCT4**) and interview (**ASCOT-INT4**) versions were the first ASCOT instruments to be developed and launched in 2012. These measures were developed through literature review, qualitative interviews and focus groups with adults accessing LTC in England [3]. The interview version (**ASCOT-INT4**) applies a novel counter-factual self-estimation [8] to estimate LTC impact, which is referred to as SCRQoL ‘gain’ [3, 9].

**Table 1:** ASCOT overview

ASCOT measure(s) †	ASCOT-SCT4/INT4 [3]	Adapted version for care homes: ASCOT-CH4 [10]	Other adapted versions: ASCOT-ER [11, 12] ASCOT-ER-OP [13] ASCOT–Proxy [14–18]	ASCOT-Carer [4, 19]	ASCOT-Workforce www.pssru.ac.uk/ascot/ascot-workforce
Measurement construct	Social care-related quality of life (SCRQoL) of adults accessing LTC services	Social care-related quality of life (SCRQoL) of adults accessing LTC services	Social care-related quality of life (SCRQoL) of adults accessing LTC services	Social care-related quality of life (SCRQoL) of unpaid (family/friend) carers	Work-related quality of life of the adult social care workforce
Target group(s)	Adults, aged 18 + years, accessing LTC services (e.g. homecare, day care, residential care) These measures are designed for use across LTC contexts, where it is possible to collect data by self-report	Residential or nursing care home residents	Adults, aged 18 + years, accessing LTC services (e.g. homecare, day care, residential care). Adapted versions are designed to support inclusion of people in research, where they are unable to complete the original version (SCT4): • ER: adults with learning disability • ER (OP): older adults unable to self-complete ASCOT, e.g., due to mild cognitive impairment, dementia • Proxy: Adults unable to self-report with help or adapted versions	Adults, aged 18 + years, who help or support another adult with care and support needs due to disability, long term health conditions or older age, unpaid	Staff working in the adult social care sector. This includes social workers, direct care roles (e.g., care worker, support worker), auxiliary or admin staff (e.g. care home cleaners or cooks), registered managers or other managers at mid-, senior or strategic levels, occupational therapists, nurses and allied health professionals
Data collection	Self-completion (SCT4) or Interview (INT4) questionnaire	Mixed methods, i.e., observation, flexible interview with residents, proxy report by care staff and family	Self-completion (SCT4) questionnaire only	Self-completion (SCT4) or Interview (INT4) questionnaire	Self-completion (SCT4) questionnaire only
Domains	• Control over daily life • Occupation (*doing things I value and enjoy*) • Social participation • Personal safety (*feeling safe*) • Food and drink • Personal comfort and cleanliness • Accommodation comfort and cleanliness • Dignity††	*See ASCOT-SCT4/INT4*	*See ASCOT-SCT4/INT4*	• Control over daily life • Occupation (*doing things I value and enjoy*) • Social participation • Personal safety (*feeling safe*) • Looking after myself • Time and space to be myself • Feeling supported and encouraged in caring role	• Making a difference • Care relationships • Autonomy at work • Time to do my job well • Worrying about work • Looking after myself at work • Safety at work • Professional relationships • Feeling supported in role • Skills and knowledge • Opportunity to develop • Income/financial security • Valued by society
UK Preference Weights available?	Yes [3]	Yes – apply self-report preference weights [3]	Yes – apply self-report preference weights [3]	Yes [19]	No

^†^ASCOT measures can be obtained for use (subject to licence) from https://www.pssru.ac.uk/ascot/

^††^There are two items for the dignity domain. These are designed to separate the effect of needing care on a person’s sense of dignity, which is not considered in the final rating, from the specific impact of *how care services are offered and delivered* on the person’s sense of dignity. The rating of the latter item feeds into the overall SCRQoL score/index

Alongside the **ASCOT-SCT4** and **ASCOT-INT4** launched in 2012 [3], a mixed methods version for care homes (ASCOT-CH3) was also released, which was later superseded by the **ASCOT-CH4** [10, 20, 21]. Since 2012, the ASCOT suite of instruments has further expanded to include versions for data collection with adults with diverse needs. These include easy-to-read versions co-produced with adults with learning disabilities (**ASCOT-ER**) [11, 12] and older adults (**ASCOT-ER-OP**) [13], as well as a proxy-report version (**ASCOT-Proxy**) for care staff or unpaid carers on behalf of people unable to self-report [14–18].

New ASCOT measures have also been added to consider wider societal perspectives of the impact of LTC*.* This includes the **ASCOT-Carer-SCT4 and -INT4**, preference-weighted measures of carers’ SCRQoL [4, 14, 19, 22–24], that are designed to measure the impact of LTC on unpaid carers’ QoL [23, 25, 26]. The **ASCOT-Workforce** has been informed by increasing awareness of the importance of the care work-related quality of life (CWRQoL) of care staff [27, 28].

Psychometric studies have found acceptable or good measurement properties for **ASCOT-SCT4/INT4** [3, 9, 24, 29–38], **ASCOT-Carer** [14, 22, 24, 39–43], **ASCOT-CH3/4** [10, 20, 21], **ASCOT-Proxy** [14, 44] and **ASCOT-ER** [11]. Some of the ASCOT measures have been translated into other languages (summarized in Table 2), with studies to establish psychometric properties and develop country-specific preference weights.

**Table 2:** Published ASCOT translations

ASCOT Measure	Language
ASCOT-SCT4	Dutch [45]; Finnish* [46, 47]; German* [48]; Japanese* [49, 50]
ASCOT-INT4	Finnish* [46, 47]; German* [48]
ASCOT-Carer SCT4	Finnish* [51]; German* [52]; Japanese* [53–55]
ASCOT-Carer INT4	Finnish* [51]; German* [52]

^†^Translations (pre-publication) are also underway for ASCOT-SCT4 into Chinese, Swedish, Norwegian, Spanish and Basque; ASCOT-Proxy into Dutch; and ASCOT-ER into Japanese

*With country-specific preference weights

In this scoping review, we aim to identify and map the use of ASCOT (any measure) in research studies. We did not consider psychometric or translation studies, briefly summarized here, but rather focus on how ASCOT has been applied. The objectives were to map ASCOT’s use in academic research to-date, the study designs within which it has been used, its contribution to concepts, and to identify any gaps for future research.

## Methods

A scoping review was conducted using the Arksey and Malley framework [56] aligned with the Joanna Briggs Institute guidelines [57]. A PRISMA-SCR checklist was completed (see supplementary file) [58].

Scoping reviews enable exploratory mapping and descriptive analysis of literature on a particular topic or area, across disciplines. They are conducted to clarify concepts, understand how research is conducted, and identify knowledge gaps [56, 57, 59]. This methodology aligns with this study’s aim of understanding and mapping academic literature, which reports use of ASCOT measures.

### Research question and objectives

In this scoping review, we consider the research question: how have ASCOT measures been used in research studies? While scoping reviews can incorporate evidence beyond research studies [57], our aim was to map existing and planned research from the international academic literature, across disciplines, to gain insight into how ASCOT has been applied in this context. Objectives were to map studies that have used ASCOT, in terms of methodology and approaches; to understand how ASCOT has informed concepts and theory through its application; and identify knowledge gaps that may be addressed by future research.

### Identifying items

The search strategy aimed to identify all academic articles reporting studies that had used one or more ASCOT measures to collect, analyze or interpret data. It was designed to be broadly inclusive, with a balance against feasibility and limiting excessive duplication.

Three databases (SCOPUS, PubMed, ProQuest), selected for breadth of coverage of health and social sciences literature, were searched on 11th July 2024 (see supplementary file). Searches were limited from 1st January 2012, to align with ASCOT-SCT4 launch timelines [3].

### Item selection and data extraction (charting)

Duplicates were removed before screening of papers by title/abstract. Where unsure, items were retained for full text screening. This initial selection was conducted by one researcher (Author1), with each record independently reviewed by one of two other researchers (Author2, Author3) to reduce bias and ensure consistency [57].

Full texts of remaining papers were considered against the inclusion/exclusion criteria (Table 3). A data chart format was developed iteratively by researchers, through data extraction [57] (see Table S1). Again, one reviewer (Author1) screened and completed the chart for included items. A second researcher completed independent checks of selection and data extraction (Author2, Author1). There was discussion to reach consensus for any discrepancies between the two reviewers, with adjudication by the third researcher, when required.

**Table 3:** Inclusion/exclusion criteria

Inclusion criteria	Exclusion criteria
Reports of research (any method, except Delphi studies) that use ASCOT measures By ‘use’, we refer to application of ASCOT in data collection, data analysis or interpretation, e.g., as a theoretical framework to guide data analysis or interpretation in qualitative studies or literature reviews	- Opinion or narrative synthesis - Delphi studies - Systematic or scoping reviews that identify and report ASCOT literature - Psychometric, content validity or development studies of ASCOT measures, including studies to elicit preference weights - Psychometric, content validity or development studies that use ASCOT as a comparator measure - Translation and cross-cultural adaptation studies for ASCOT measures - Studies that used measures other than ASCOT, including where these measures are (mistakenly) referenced as ASCOT - Studies that apply early developmental versions of ASCOT-SCT4 (e.g., ASCOT-SCT3, OPUS) - Studies of the developmental version of ASCOT-SCT4, pre-2012
Research conducted in any LTC settings or context, with adults aged 18 years or over	Research conducted with children or young people, aged under 18 years
Published in peer-reviewed journal articles, books or book chapters, including study protocols, where ASCOT use was not reported in an article identified by our searches	- Grey literature (e.g. policy documents, reports) - Conference proceedings - Protocols, where ASCOT use from the study is reported in an included article
Published in English or publicly-available in English translation	N/A
Any geographic region or country	N/A
Published since 2012, until date of search	N/A

### Summarizing and synthesizing

Selected items were organized and reported descriptively [57], organized by the study designs within which ASCOT measures have been applied, to align with the research question and study objectives.

## Results

**Fig. 1:**
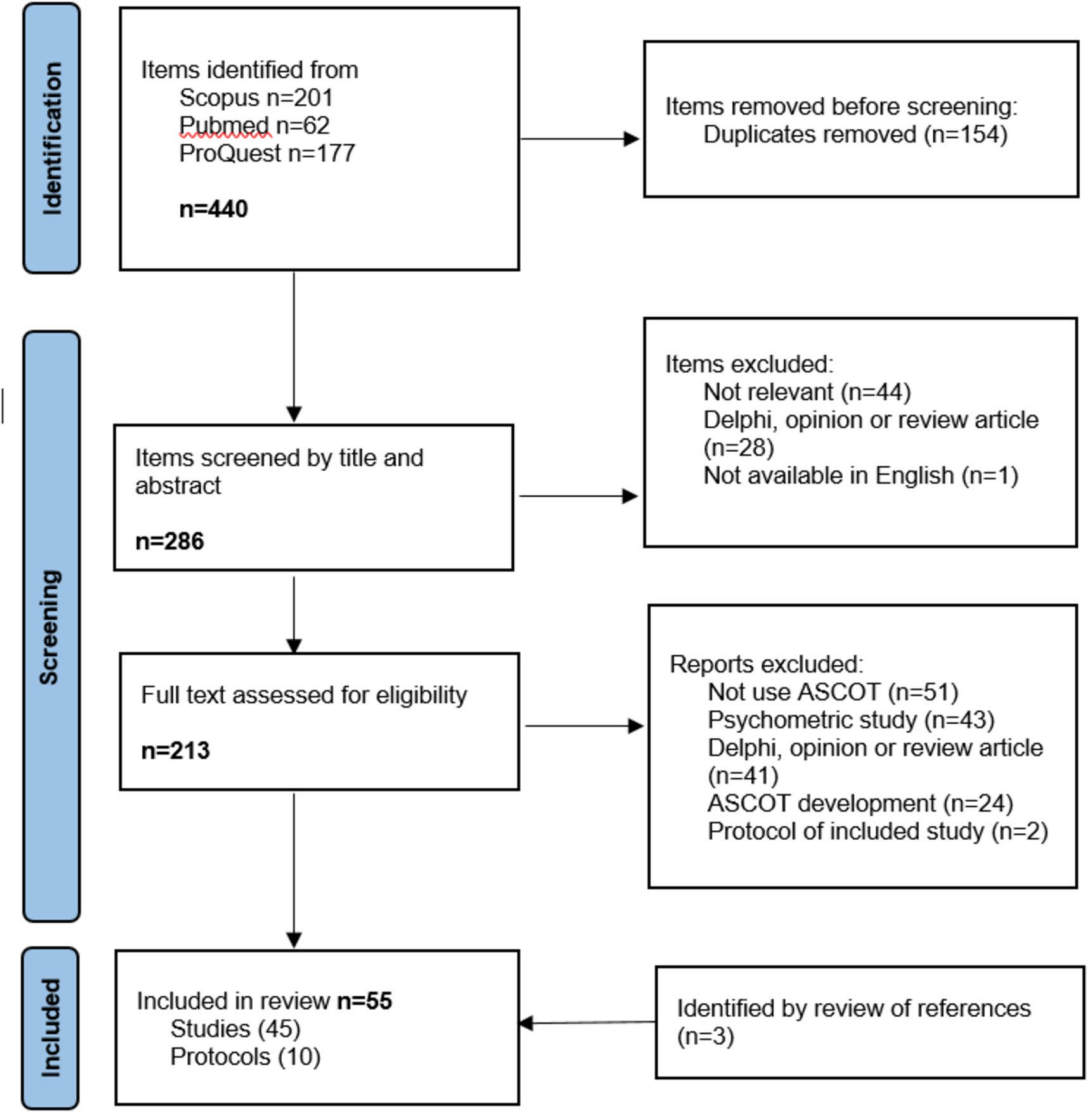
Flow diagram

A total of 55 articles were identified (Fig. 1), of which eleven were protocols. Eleven articles reported sub-studies from the following four studies: Identifying the Impact of Adult Social Care (IIASC) [23, 26, 61, 69, 70], Exploring Comparative Effectiveness and Efficiency in Long-term Care (EXCELC) [63, 75], Measuring the Outcomes of Care Homes (MOOCH) [72, 74] and *FindMyApps* RCT [82, 83].

Most studies were conducted or planned in the UK (n = 38, see Table S1). Other studies were in Australia (n = 6) [79, 85, 92, 100, 102, 109], the Netherlands (n = 5) [82–84, 87, 88], Finland (n = 2) [73, 101], Austria (n = 1) [80] and Germany (n = 1) [95]. Two articles reported on different aspects of a comparative study of the UK, Austria and Finland [63, 75]. This geographic distribution of studies reflects the current availability of ASCOT translations into English, Dutch, German and Finnish. No Japanese studies were identified, despite available Japanese translations [112, 113].

Most articles used **ASCOT-SCT4** (n = 28) or **ASCOT-INT4** (n = 8, see Table S1). Ten studies used **ASCOT-Carer-SCT4** or **ASCOT-Carer-INT4**, with seven studies of **ASCOT-CH3/4** [10, 67, 72, 74, 100, 103, 110] and two of **ASCOT-Proxy** [71, 104]. No studies used **ASCOT-ER**, but one study analyzed data collected from adults with learning disabilities using an informal adaptation (non-validated) [68]. Another study developed an informal proxy-report version, which pre-dates the ASCOT-Proxy developed officially by ASCOT developers, to collect proxy report data from family carers and care staff supporting adults with learning disabilities [105]. One protocol reported the planned use of ASCOT, but did not state the version [78].

The relative use of ASCOT measures may partially reflect the chronology of their release. **ASCOT-SCT4/INT4** and **ASCOT-CH3** (later, CH4) was launched in 2012, followed by **ASCOT-Carer-SCT4/INT4** in 2015, **ASCOT-ER** in 2017, **ASCOT-Proxy** in 2022, and **ASCOT-ER-OP** and **ASCOT-Workforce** in 2024. Limited use of **ASCOT-ER** and **ASCOT-CH3/4** are notable. This may be partly due to barriers to people with learning disabilities participating in research [114, 115] and the resource intensity of **ASCOT-CH3/4** data collection [10], respectively.

Selected papers were categorized into cross-sectional observational (*n* = 19), randomized control trial (RCTs) (*n* = 15), mixed method (*n* = 14), and other designs (*n* = 7).

## Cross-sectional/observational

Nineteen articles reported cross-sectional studies that applied ASCOT as an outcome measure in quantitative analysis to address a range of LTC practice and policy-relevant questions.

Six studies applied econometric techniques to estimate effectiveness of specific interventions, policy or system-level LTC [61, 62, 64–66, 77]. Experimental designs to limit bias in estimation of impact (e.g., RCTs) are often not feasible in LTC studies, especially for care provided on a statutory basis. Instead, various analytical strategies, including instrumental variables (IV), production function or propensity score matching [61, 62, 64–66, 77], enable estimation of the impact of the intervention on outcomes, controlling for selection bias and other limitations of cross-sectional designs that apply regression (e.g. unobserved variables, endogeneity, attribution bias).

The remaining articles (*n* = 13) reported regression analyses, which are more susceptible to the issues outlined above, but may still provide useful insights. These studies tended to focus on the relationship between SCRQoL (as a key outcome or indicator of quality of LTC) and other relevant factors, and to explore the implications for policy and practice. Four articles applied **ASCOT-CH3/4** to measure QoL of older care home residents [10, 67, 72, 74]. Of these, three explored whether care home residents’ SCRQoL was associated with care quality rating by the UK care regulator, with evidence of an association [10, 67, 74]. The fourth article found that resident SCRQoL was lower out-of-hours, which was used to argue for greater consideration of support outside of office-hours [72].

Eight of the 14 articles reporting regression analysis were studies of adults living at home and/or their family carers [63, 68–71, 73, 75, 76], with a further study across care settings [60]. One study sought to establish whether carers’ SCRQoL is related to their self-reported reason for caring, which pertains to UK and international policy discussions around the importance of choice and control for carers [70]. Other papers sought to identify the factors related to SCRQoL for older adults using publicly-funded services [76], adults with learning disabilities using publicly-funded services [68] and people with dementia and their carers engaging with services, whether self- or publicly-funded [71], as relevant to policy and practice. These analyses highlighted the contribution of housing quality and suitability for individual needs, financial status or security, and health status on QoL, which points to the need for better public service integration and welfare to support outcomes [68, 71, 76].

Two articles from a single study [63, 75] applied **ASCOT-INT4** and **ASCOT-Carer-INT4** in a cross-country comparative analysis of the performance of homecare for older people in England (UK), Austria and Finland. The analysis found a significant association between socio-economic status and QoL gain (i.e., impact of services on QoL) in England, but not Austria or Finland, which indicates that the English care system is less effective at supporting older people, across socio-economic status [75]. By contrast, the analysis of carer SCRQoL did not find any significant differences by country, although English carers were more likely to be co-resident and report poorer health than those in Austria or Finland [63].

Across these studies, there were examples of novel analytical or conceptual applications of ASCOT. These include the production function approach to estimate the impact of LTC on SCRQoL [61, 62], using the INT4 method to understand to the effect of client choice on SCRQoL outcomes of homecare users [73], and comparative studies of by-country differences in SCRQoL gain (i.e., impact or effect of services) [75]. In addition, one article adopted a novel ‘dyadic’ analytical approach. The Actor Partner Interdependence Model (APIM) was applied to **ASCOT-SCT4** and **ASCOT-Carer-SCT4**, to demonstrate the interdependence of SCRQoL between individuals in caregiving relationships, especially for *Control over daily life* [69]. Finally, some studies applied ASCOT to conceptualize ‘unmet LTC need’ in relation to SCRQoL, rather than functional impairment, strain or burden [71].

## Randomized control trials (RCTs)

Fifteen articles reported full or feasibility RCTs to demonstrate the effectiveness of LTC interventions with ASCOT as a primary or secondary outcome measures (see Table S1). Studies were of older adults, living at home or in care homes, except for one study of homecare workers [80] and another of people living in vulnerable households [79].

The studies evaluated a range of LTC ‘innovations’ in care delivery or organization, e.g., educational intervention for care workers [81, 85], apps designed to support care-delivery [80, 82–84, 92], routine COVID screening in residential care [78], comprehensive geriatric assessment of older adults living with HIV [86], occupational therapy in reablement services [91], and exercise designed to reduce falls and improve cognitive status [87, 88]. ASCOT has also been used in England to evaluate the well-established LTC intervention of bathroom adaptations for mobility-impaired older adults [89, 90]. Another study explored an intervention outside of what is traditionally understood by LTC, but is closely-related – namely, housing [79].

Issues related to the selection of outcome measures in RCTs were evident in some cases. For example, the German-language **ASCOT-Carer-SCT4** [80] was used in one study to understand the effect of a workplace software intervention on care work-related quality of life (CWRQoL) of care workers [80], even though **ASCOT-Carer-SCT4/INT4** were developed to measure family carer SCRQoL [4, 116]. It was not developed to capture CWRQoL, and is unlikely to have been sensitive to the effect of intervention.

Also, three articles reported use of single **ASCOT-SCT4** item selected from the instrument, rather than applying the full measure [84, 85, 88]. In the two completed studies, there was no significant effect of intervention on the ASCOT item outcome [84, 88]. This may be partly due to the limited rating range and variation of single ASCOT items (1 to 4, Likert-like scale), by comparison to the overall **ASCOT-SCT4** preference weighted index (-0.17 to 1.00, continuous scale).

## Mixed methods

Fourteen articles reported mixed method studies (see Table S1). The majority of these (n = 9) evaluated the costs and/or outcomes of LTC interventions, including: reablement for older adults with dementia [93]; staffing intervention in nursing homes [95]; care by micro care providers [97]; specialist nursing intervention for dementia carers [98]; circles of support for adults with intellectual disabilities [105]; active management of QoL for people with dementia [96]; culture change programme in residential care homes [100]; and day activities for older adults [101, 102]. These applications of ASCOT align with the design and intended use of ASCOT to evaluate LTC interventions, broadly, across contexts and settings.

Three of the five remaining studies reported descriptive mixed methods analysis of older people using day centers’ QoL [99], carers’ QoL to develop a theoretical framework for the impact of LTC on QoL [26], and older adult care home residents’ QoL to explore ‘meaningful activity’ in residential care [72]. These applications illustrate how ASCOT can be used to generate descriptive findings, to explore and refine theoretical concepts related to the quality, effectiveness and outcomes of LTC.

The last two articles were protocols for a mixed methods descriptive study of the QoL impact of COVID outbreaks on care home residents [94] and the pilot of a minimum dataset for UK care homes, which used **ASCOT-Proxy** due to the challenges of collecting self-report SCRQoL from care home residents [104]. The latter illustrates the use of ASCOT in routine data collection to inform individual assessment and care planning, quality monitoring and improvement in service delivery, and analysis at aggregate level to inform policy and planning.

Unlike RCTs, which mostly applied ASCOT–SCT4, only five of the mixed methods articles used **ASCOT-SCT4** [93, 95, 96, 102] or **ASCOT-Carer-SCT4** [98]. Five articles applied **ASCOT-INT4** [97, 99, 101, 105] or **ASCOT-Carer-INT4** [26] to estimate SCRQoL gain, a measure of the impact of LTC using counterfactual self-estimation [106], which is unsurprising given the INT4 methodology lends itself to studies using mixed methods.

## Other designs

Seven articles applied other study designs (see Table S1). Three studies were quantitative studies to evaluate the costs and/or outcomes of LTC interventions – specifically, pre-post test design to evaluate an information signposting service for carers [109], a cost consequence study of older adults using a help-at-home scheme [111], and a retrospective comparative study of older adults using direct payments [107]. As with RCTs, observational or mixed methods studies that evaluated interventions, these studies used the **ASCOT-SCT4** or **ASCOT-Carer-SCT4**.

One of the four remaining studies was an exploratory study of implementing ASCOT feedback in older adult care homes to guide care delivery and planning by staff, in which the intervention was acceptable to staff, even if use of **ASCOT-CH3** routinely may not be feasible due to the resource intensity of data collection [110]. Another study applied **ASCOT-SCT4** as a theoretical framework in a scoping review of QoL impacts of digital engagement among older adults [108].

Two qualitative studies explored the experience and impact of LTC on carers’ SCRQoL [23] and bathing adaptations for older adults and their carers [117], using **ASCOT-Carer-INT4** and **ASCOT-SCT4**, respectively. In one study [23], **ASCOT-Carer-INT4** was used to structure the interview topic guide and informed the analysis and interpretation, which identified the theme of who services are ‘for’, which relates to how carers are recognized and engaged by LTC services. In the second study [117], ASCOT-SCT4 data were collected alongside interviews, with data analysis and interpretation that ‘read across’ between the qualitative and quantitative data.

## Discussion

The ASCOT instruments are suitable for use as outcome measures in LTC (economic) evaluation and research, across settings, contexts and client need or age group [5, 6, 30, 118]. This scoping literature review contributes to the evidence base by mapping the use of ASCOT, across 55 articles, since its release in 2012. The majority of identified studies, across designs, applied the **ASCOT-SCT4** in (economic) evaluation, its original intended use [3]. This is unsurprising because the **ASCOT-SCT4** was designed for breadth of use by adults, aged 18 or over, in different LTC settings. It has also been available with preference weights for over a decade.

**ASCOT-Carer** also has preference weights, but it has not yet been as widely-used. This may be due to the later date of release (i.e., 2019 [19]). The consideration of unpaid carer outcomes in LTC evaluation is less well-established and subject to methodological debate [23, 25, 26]. Preference weights are not yet available for adapted versions of the **ASCOT-SCT4** designed to support accessibility (i.e., **ASCOT-ER, -Proxy, -ER (OP)**) or the **ASCOT-Workforce**, released in 2024. This may affect their use in evaluation studies. Future research would usefully establish whether version-specific preference weights are justified for the adapted versions of the ASCOT-SCT4, and to generate preference weights for the ASCOT-Workforce.

Even if preference weights were available, relatively low uptake of ASCOT-ER, despite its advantages in improving accessibility, may persist. This is because of wider barriers to research participation for adults with learning disabilities (the target group for use of ASCOT-ER), as well as an associated move away from use of structured questions, even if adapted to support participation, in favour of flexible and emancipatory methods [114]. A direction for future research is further exploration of how to better enable and facilitate participation of adults with learning disabilities in LTC (economic) evaluation studies.

A number of studies have developed translations (e.g., [119, 120]), which support the use of ASCOT in evaluation studies, internationally, within countries, as well as in cross-country comparative policy research to evaluate the relative performance of LTC systems (e.g., [63]). We expect international use of ASCOT measures to expand as new translations are developed and time passes from date-of-release for existing translations, to allow the opportunity for measures to be applied in research. The uptake and application of translated versions of ASCOT may, however, be affected by the degree of policy focus, skills and expertise of the research workforce, and availability of funding in LTC research, both within and between countries.

In the identified studies of this scoping review, we find examples of common issues in the application of QoL outcome measures in evaluation studies that are not unique to ASCOT, but they nevertheless important to consider. First, the example of ASCOT-Carer applied to measure care workers’ QoL, when it was developed as a measure of SCRQoL for unpaid carers [80]), highlights the need for careful selection of outcome measure. It is important to ensure selected measure(s) are suitable for the study setting, context, population and aims. Second, the use of a single item from a measure may enable a focus on a particular domain (e.g., [84, 85, 88]), but it may affect sensitivity, since the full measure is not scored or applied, and precludes any form of cost(-effectiveness) analysis using preference weights.

Our findings also identify that ASCOT has been used in ways that contribute to the development of theory and concepts in LTC. In qualitative studies [61] and scoping reviews [108], for example, ASCOT has been used to structure analysis and develop interpretation. Theoretical frameworks using ASCOT have also been used to develop and inform empirical analysis, such as dyadic analysis of SCRQoL to understand QoL interdependence in caregiving dyads (e.g., [69], as developed in subsequent literature [121, 122]). ASCOT measures have also been used to develop concepts and theory of how LTC services affect people’s QoL, as well as how to conceptualise unmet needs, with regard to QoL (e.g., [71]).

The findings of this review also illustrate how the use of ASCOT has potential to drive innovation and inform development of LTC policy and practice. For example, **ASCOT-SCT4** is collected routinely in the English Adult Social Care Survey and is available for policy analysis by researchers. These data were used in a number of studies identified here (e.g., [66, 68]). **ASCOT-Proxy** has also been included in a UK pilot minimum dataset (MDS) for older adult care homes, to develop a dataset that links health and LTC data from existing sources, including data collected by care homes. The inclusion of ASCOT-Proxy, alongside other resident-level QoL measures, was informed by a drive to highlight resident QoL as a key outcome of care and indicator of quality, safety and effectiveness [104]. Implementation of **ASCOT-Proxy** in a routine UK MDS, linking QoL to other health and care data at an individual level, including workforce data, could provide a rich dataset for national or regional-level analysis, to inform decision-making, policy development and planning.

A notable gap in the identified literature is the use of ASCOT in care planning, assessment and local service delivery or planning. This scoping review may not have fully captured such literature, due to its focus on academic journals. Such use may be better reported in practice publications or reports. In academic journal articles on the use of QoL outcome measures (like ASCOT) in LTC practice, especially by LTC providers, however, the relative paucity of evidence from practice is likewise noted [121, 123], along with the barriers and challenges that may affect implementation–e.g. limited resources for data collection, analysis and interpretation, despite pressure from funders and commissioners to evidence impact [124]. The **ASCOT-INT4** may offer a pragmatic way to evidence outcomes for LTC providers; indeed, two of the identified studies suggest this as a possibility [99, 102]. Therefore, implementation of ASCOT in care practice is a potential direction and focus for future research.

The study has some limitations. Notably, we focussed on published academic literature only, since our focus was to map and understand use in academic studies. There has been use of ASCOT in policy research and practice, which may not have been captured in our review, due to restricted reporting and publication. To further consider application of ASCOT, especially in care delivery and policy analysis within organisations (e.g. local authorities, government departments), we would recommend a separate study using different methods (e.g., qualitative methods, case study, document analysis). Second, we limited our study selection to English language publications, which may have contributed to the absence of Japanese studies, despite there being Japanese translations. As a mitigation, the Japanese ASCOT translation team were asked to report any known studies using Japanese versions of ASCOT; they were not aware of any such academic studies, which aligns with our findings.

Despite these limitations, we present a comprehensive review of ASCOT use in academic research since 2012, which maps the use of ASCOT in LTC (economic) evaluation and its contribution to policy and practice-relevant research. ASCOT research has focussed on (economic) evaluation studies of LTC policy and interventions, but the measures have also contributed to advance LTC theory and concepts, e.g., in shaping concepts around unmet care needs and the impact of LTC, especially to adopt a wider perspective to include also unpaid carers and the workforce. Future directions for research include studies of ASCOT implementation in care practice, including care planning, delivery and evidencing of impact to guide decision-making. Further development (especially translation into new languages and elicitation of preference weights for ASCOT-Workforce) would support the application of ASCOT to further the LTC evidence base, both in the UK and internationally.

## Supplementary Information

Below is the link to the electronic supplementary material.

Supplementary file1 (PDF 38 KB)

## Data Availability

N/A. This is a scoping review.
