# Supplementary material for: Use of the adult social care outcomes toolkit (ASCOT) in research studies: an international scoping review
Source: Qual Life Res. 2025 Apr 18;34(9):2437–50. doi: 10.1007/s11136-025-03958-3 (PMC12432070; doi:10.1007/s11136-025-03958-3)
Supplement: Supplementary file 1 — Supplementary file1 (PDF 38 KB) [file 11136_2025_3958_MOESM1_ESM.pdf]

Table S1 Data extraction of selected items by study design (observational, RCT, mixed methods and other designs)

**Authors, year [References]**	**Study design**	**Study or protocol?**	**Single paper per study?**	**ASCOT measure (item)**	**Country**	**Sample description**	**Sample size**	**Summary of study purpose and key findings relevant to use of ASCOT**
Callaghan & Towers, 2014 [89]	Observational	Study	Yes	SCT4 (Control)	UK	Older adults in extra care housing<br>… care homes<br>… using homecare	102<br>215<br>301	A secondary analysis of cross-sectional data collected across four studies of older adults living in different settings (extra care housing, care home, and at home) to examine the association between control over daily life and care setting. After controlling for individual characteristics of age, ability activities of daily living and self-rated health in an ordered logistic regression, care homes and extra care housing residents reported similar ASCOT-SCT4 *Control over daily lifl*; older people receiving care at home reported lower ASCOT-SCT4 *Control over daily life.*
Forder et al, 2018 [49]	Observational	Study	No (IIASC)	SCT4	UK	Using homecare	622	An econometric analysis that applied a 'production function' approach with the aim of modelling the association between service utilisation and ASCOT-SCT4 SCRQoL. It was found that community long-term care significantly improved people's ASCOT-SCT4 SCRQoL but with diminishing marginal effects. Effects were also differentiated by baseline impairment levels.
Forder et al, 2014 [87]	Observational	Study	Yes	SCT4	UK	Older adults using homecare	301	A cross-sectional 'proof of concept' study to apply a production function approach to estimate the impact of homecare services on older adults' ASCOT-SCT4 SCRQoL. This applied instrumental variables (IV), which were in this case 'type of local authority'. The method (with limitations, as discussed) was able to estimate the marginal impact of homecare.
Linnosmaa et al, 2024 [53]	Observational	Study	No (EXCELC)	Carer INT4	UK, Austria, Finland	Unpaid carers	835	A comparative study of ASCOT-Carer SCRQoL to compare carers' QoL in England (UK), Austria and Finland. The study found that English carers were more likely to be co-resident with the person they care for and report poor health. They also had significantly higher expected SCRQoL (i.e. in the absence of services), which may partly be explained by the differences in sampling by country. After risk adjustment and multiple imputation to deal with missing values, none of the between country differences were statistically significant in regression models.
Longo et al, 2019 [86]	Observational	Study	Yes	Carer SCT4	UK	Unpaid carers of people with dementia living at home	330	A cross-sectional feasibility study to evaluate the economic impact of Admiral Nursing on carers. Three econometric techniques were applied to compare costs and outcomes with/out AN, whilst controlling for observed and unobserved factors: linear regression (with/out SE clustering by local authority), propensity score matching and instrumental variables (IV). Outcomes included ASCOT-Carer SCRQoL, carer self-efficacy for managing dementia (SEMD) and subjective wellbeing on a 10-point scale. Across the three methods, after controlling for other factors, the outcomes of AN carers were found to be the same or slightly better than non-AN carers. Costs were similar between the two groups.
Longo et al, 2020 [85]	Observational	Study	Yes	SCT4	UK	Adults using vision rehabilitation services	230	An economic evaluation of in-house contract vision rehabilitation (VR) services using two perspectives (1) social care alone and (2) health and social care combined. Outcomes ASCOT-SCT4, EQ-5D) and resource/cost data were collected through telephone interview survey at baseline and follow-up at M1, 2 and 6. Individual characteristics were collected at baseline only. The analysis also considered LA-level factors (demand and supply side), alongside user-level (demand side), since LA policy may also influence availability or use of in-house vs contracted-out VR. Regression analysis using generalised least square (GLS) random effects, with clustering of SE by local authority. Multiple imputation was applied to deal with missing data. Cost effectiveness was established using incremental net health benefit INHB). The analysis found that in-house services had a higher probability of being cost-effective than contracted-out services from a social care perspective. Adopting a health and social care perspective, however, in-house services were less likely to be cost effective.
Longo et al, 2021 [84]	Observational	Study	Yes	SCT4	UK	Adults using publicly-funded care	52,607	A cross-sectional study to estimate the impact of public long-term care expenditure on quality of life. Using ASCOT-SCT4 data collected in the 2017/18 Adult Social Care Survey (ASCS), on public expenditure and local authority characteristics, an instrumental variable approach was applied to estimate the impact of expenditure on service users' ASCOT-SCT4 SCRQoL. These results suggest that public ASC is effective in increasing users' quality of life but only to a relatively small extent.

Authors, year [References]	Study design	Study or protocol?	Single paper per study?	ASCOT measure (item)	Country	Sample description	Sample size	Summary of study purpose and key findings relevant to use of ASCOT
Netten et al, 2012 [74]	Observational	Study	Yes	CH3	UK	Older adults resident in care homes	366	A cross-sectional study of the ASCOT-CH3 SCRQoL of older adults resident in care homes, to determine if QoL is associated with quality ratings allocated by the UK care regulator, the Care Quality Commission (CQC). A random intercept multilevel model was applied to model ASCOT-CH3 SCRQoL gain. The model took into account individual resident factors, variation by fieldwork rater and other home-level factors (e.g. time in business). It was found that ASCOT SCRQoL gain was related to CQC quality rating for residential homes, but not for care homes.
Rand & Malley, 2017 [78]	Observational	Study	Yes	ER*<br><br>**Informal adaptation*	UK	Adults with learning disabilities using community-based social care	13,642	A cross-sectional analysis of two waves (2011, 2012) of the Adult Social Care Survey (ASCS) in England to explore factors related to ASCOT (informal ER adaptation) SCRQoL and changes over time. Regression (OLS) analysis found variation by local authority and over time, with better outcomes in 2012 vs. 2011. Poor home design, self-rated health, being male and proxy response were related to lower SCRQoL, whereas being over 65 years and white ethnicity were related to better SCRQoL. Analyses were also conducted by each ASCOT domain using GOLOGIT models. Improved QoL between 2011 and 2012 were observed in five SCRQoL domains, after controlling for other factors: *Personal comfort and cleanliness*, *Personal safety*, *Social participation*, *Occupation* and *Dignity*.
Rand et al, 2017 [51]	Observational	Study	No (IIASC)	INT4, Carer INT4	UK	Dyads (i.e. matched pairs) of adults using social care due to physical disability or mental health needs and their carers	264	A cross-sectional study to examine the interdependency of SCRQoL for care-recipients and their carers. This study applied dyadic analysis using the Actor Partner Interdependence Model, operationalised as a multilevel mixed effect ordered logistic regression, to the three ASCOT domains that are in both the ASCOT-SCT4 and ASCOT-Carer SCT4: *Control over daily life*, *Social participation* and *Occupatio*n. A 'partner effect' was found for *Control over daily life*, whereby better rating of satisfaction with services by care-recipients was associated with higher SCRQoL for carers. This partner effect was not observed for the other two domains, but various individual and contextual factors were found to affect each person's SCRQoL, either as actor effects (i.e. an individual's characteristics affecting their own QoL) or partner effect (i.e., the care dyad partner's characteristic affecting the person's outcome). There was also unobserved interdependence (i.e. direct influence of Control of one person on the other) for *Control over daily life*, but not the other two domains.
Rand et al, 2018 [50]	Observational	Study	No (IIASC)	Carer INT4	UK	Unpaid carers of adults using community-based social care	387	A cross-sectional study to explore whether self-reported reasons for caring are associated with carers' ASCOT-Carer SCRQoL and also carer strain. In regression analysis, the reported ‘reasons for caring’ were related to both carer SCRQoL and strain. Where the reason was reported as (1) because social services suggested it or (2) the care-recipient would not want help from anyone else, carers were more likely to report lower SCRQoL. Where carers (3) took on care-giving because they had time to care, this was significantly associated with better SCRQoL.
Rand et al, 2022 [77]	Observational	Study	Yes	Proxy, Carer SCT4	UK	Unpaid carers of people with dementia living at home, who use community-based social care	313	A cross sectional study to examine the factors associated with ASCOT-Carer SCRQoL for carers and the people they support (ASCOT-Proxy SCRQoL, proxy report). Multiple regression found that factors significantly related to carers’ SCRQoL were their health, financial difficulties associated with caring, and satisfaction with social care support. Inadequate home design was significantly negatively associated with proxy-reported SCRQoL. The latter stages of the pandemic-related restrictions (UK tier system from 2nd December 2020 to study end, April 2021) were associated with significantly worse SCRQoL for people with dementia, but not carers. The study also described 'unmet need' based on the rating of SCRQoL by domain, which showed the high level of unmet need in the sample of dementia carers, especially in higher order domains (>50% of sampled carers reported unmet need in five of the seven ASCOT-Carer domains, with the exception of *Self-care* (32.1%) and *Personal safety* (3.2%)).
Smith et al, 2019 [54]	Observational	Study	No (MOOCH)	CH4	UK	Older adults resident in care homes	99	A cross-sectional study of ASCOT-CH4 SCRQoL of older adults resident in care homes to determine if there are differences between office hours (9:00 to 16:30) and out-of-hours in the evening (16:30 to 20:00) or weekends. ASCOT-CH4 SCRQoL was measured at two time points, within and out-of-hours. Ratings were compared by within or out-of-hours, using parametric (independent samples t-test) and non-parametric (Mann-Whitney U test) analysis,

Authors, year [References]	Study design	Study or protocol?	Single paper per study?	ASCOT measure (item)	Country	Sample description	Sample size	Summary of study purpose and key findings relevant to use of ASCOT
								as well as general linear model (GLM) controlling for covariates (care home). This exploratory study found that residents had worse SCRQoL out-of-hours than between office hours.
Steffansson et al, 2016 [68]	Observational	Study	Yes	INT4	Finland	Older adults using homecare	2,096	A cross sectional study of older adults using homecare in Finland, to understand the association between 'freedom of choice' and the effectiveness (measured using ASCOT-INT4 SCRQoL 'gain') of homecare. Multivariate regression analysis with ASCOT-INT4 SCRQoL gain found significant associations with freedom of choice with regard to: meals/food, time of care worker visit and day for house cleaning.
Towers et al, 2021 [12]	Observational	Study	Yes	CH4	UK	Older adults resident in care homes	475	A psychometric study (not considered further in this review) followed by cross sectional analysis of ASCOT-CH4 data to establish the association between SCRQoL and care quality rating by the English care regulator, the Care Quality Commission (CQC). Regression analysis found no significant association between ASCOT-CH4 SCRQoL 'basic' domains and care quality rating, which indicates all homes are able to address basic needs. However, there were significant differences for higher order ASCOT-CH4 domains, especially *Control over daily life* and *Social participation*. Of the CQC ratings in the five domains of care quality, the domains of 'caring' and 'well led' were more strongly associated with SCRQoL than other domains. 'Well led' was the only domain of the five CQC domains associated ASCOT-CH4 basic domains, especially for *Personal safety* and *Food and drink*.
Towers et al, 2019 [55]	Observational	Study	No (MOOCH)	CH3	UK	Older adults resident in care homes	266	A cross-sectional study of ASCOT-CH3 SCRQoL using Multilevel modelling (MLM) to explore whether SCRQoL was associated with ratings of care quality by the CQC. The random intercept MLM indicated that better SCRQoL was associated with individual (being female, better functioning, no dementia diagnosis and fewer communication issues) and also care quality (rated as good/outstanding vs. requires improvement) factors. The size and registration category (residential or nursing) were not significantly associated with SCRQoL.
Trukeschitz et al, 2021 [52]	Observational	Study	No (EXCELC)	INT4	UK, Austria, Finland	Older adults using homecare	811	A comparative study of ASCOT-INT4 SCRQoL in England (UK), Austria and Finland to establish the factors associated with ASCOT-INT4 SCRQoL gain (i.e. counter-factual estimation of impact of services on SCRQoL). Across all three countries, SCRQoL gain (impact of services) increased with need. Also better quality care process was associated with higher SCRQoL gain, across all three countries. Homecare improved service users' QoL, with marginal effects reduced at high care intensity, across all three countries. This indicates that homecare is able to address people living at home, but is more limited when seeking to compensate for very high needs.
van Leeuwen et al, 2014 [88]	Observational	Study	Yes	SCT4	UK	Older adults living at home using care services	29,935	A cross-sectional study of ASCOT-SCT4 data collected in the English Adult Social Care Survey (ASCS) in 2010/11 to explore factors related to SCRQoL. Regression analysis found that SCRQoL is significantly lower for those who find it hard to access information about care, those who report their home is inappropriate for needs and whose local area is less accessible.
Yang et al, 2017 [83]	Observational	Study	Yes	SCT4	UK	Adult Social Care Survey sample for 2010, 2011 and 2012, aged 65+ in residential care	Not reported	A study that applied a novel approach to establish productivity in long-term care that adjusts for service users' outcomes using ASCOT-SCT4 collected in the English Adult Social Care Survey (ASCS). The analysis applied a cost-weighted output index with quality adjustment using ASCOT-SCT4 SCRQoL and an input index based on local authority adult social care spend, to then calculate productivity. The analysis showed that it is feasible to calculate adjusted productivity ratios for older people in England in residential or nursing care. The findings were a productivity increase from 2010 to 2011, with no change from 2011 to 2012.

Authors, year [References]	Study design	Study or protocol?	Single paper per study?	ASCOT measure (item)	Country	Sample description	Sample size	Summary of study purpose and key findings relevant to use of ASCOT
Adams et al 2023 [80]	RCT	Protocol	Yes	Not stated	UK	Older adults living in residential or nursing care homes	Residents of 280 care homes	A pragmatic cluster RCT to evaluate asymptomatic COVID testing compared with standard care in older adult residential or nursing care. 280 care homes will be randomised into the study. The primary outcome is COVID-related hospitalisation. Secondary outcomes will include the number and duration of COVID outbreaks and closures. Process evaluation will be conducted by qualitative interviews (n=28 care homes). Following collection or primary/secondary outcomes and process evaluation, six homes will remain in the study to participate in ASCOT interviews to explore QoL impacts on residents. No detail is provided on how these interviews will be conducted or which measure(s) will be used.
Campbell et al, 2022 [59]	RCT	Protocol	Yes	SCT4	Australia	Vulnerable households	1,000	A staggered parallel group RCT to evaluate the effectiveness of the Victorian Healthy Homes Program (VHHP) to upgrade homes to improve thermal comfort, reduce energy use and to provide health and economic benefits. Primary outcome is the average difference in home temperature between intervention and control, over winter. ASCOT-SCT4 is included as one of three (also, EQ-5D and SF-12) individual-level health-related quality of life outcome measures.
Haslinger-Baumann et al, 2023 [69]	RCT	Study	Yes	Carer SCT4	Austria	Homecare workers delivering 24h care	110	A parallel three-arm study design (control, partial and full intervention) was used to evaluate a software solution to support quality assurance of 24-hour homecare. The primary outcomes were ASCOT-Carer SCT4 SCRQoL of the paid care workers and their self-efficacy. No statistically significant difference overall or by domain was observed for ASCOT-Carer at follow-up 1 (five months). At nine-month follow-up, applying intention to treat, care workers in the intervention group reported significantly better outcomes. The ASCOT-Carer SCT4 *Feeling encouraged and supported* domain was rated significantly better than control ($p<0.01$).
Kinderman et al, 2018 [90]	RCT	Study	Yes	SCT4*<br><br>**Self report or by proxy*	UK	People with dementia living on inpatient wards and in care homes	439	A clustered randomised design to evaluate a human rights approach intervention (training and assessment) at 10 intervention and 10 control sites. ASCOT-SCT4 was used alongside EQ-5D-3L for the economics component. The reporting of the health economics element of the study was limited, with only partial ASCOT-SCT4 data completed. Of these, responses were split by self- and proxy-report, adding to the limitations. However, the average ASCOT-SCT4 SCRQoL was higher in both the self-completed and proxy-reported data for intervention vs. control groups.
Neal et al, 2023 [56]	RCT	Study	No (trial ref.: NL8157)	SCT4	Netherlands	Dyads of people with mild dementia or MCI and their carers, living at home	128	A single-centre, two-arm non-blinded RCT to evaluate FindMyApps, an eHealth intervention to support tablet use and identification of easy-to-use apps. No significant difference was found between intervention and control groups at 3-month follow-up for the primary outcomes of ASCOT-SCT4 SCRQoL and Maastricht Social Participation Profile frequency and diversity scores.
Neal et al, 2024 [57]	RCT	Study	No (trial ref.: NL8157)	SCT4	Netherlands	Dyads of people with mild dementia or mild cognitive impairment (MCI) and their carers, living at home	150	A cost effectiveness analysis of data collected from all 150 dyads in the RCT reported in Neal et al, 2023 (see above). No significant difference of outcomes (EQ-5D-3L, ASCOT-SCT4) was found for the intervention compared to control group for people with mild dementia or MCI. There was a significant difference for the carer outcome of sense of competence. For all outcomes, the probability that FindMyApps was cost-effective at a willingness-to-pay threshold of €0 per point of improvement was 0.72 for people with dementia and 0.93 for caregivers.
Beentjes et al, 2023 [64]	RCT	Study	Yes (trial ref.: NL7210)	SCT4 (*Social*)	Netherlands	Dyads of people with mild dementia or MCI and their carers, living at home	59	An exploratory RCT to evaluate the impact of FindMyApps for people with dementia/MCI and their carers. Various outcome and experience measures were considered, including a single item from ASCOT-SCT4 (*Social participation*). After three months, no significant difference on any of the selected measures - including ASCOT-SCT4 social participation - were observed between intervention and control group.
Savvas et al, 2021 [60]	RCT	Protocol	Yes	SCT4 (*Social*, *Control*)	Australia	People living at home with dementia, their carers and homecare workers	108 PwD & carers<br>216 HCWs	A stepped-wedge cluster-RCT to evaluate a dementia education and upskilling programme. The primary outcome is sense of competence reported by homecare workers on the Sense of Competency in Dementia Care Staff questionnaire (SCID-S). Secondary outcomes include ASCOT *Control over daily life* and *Social participation*.

**Authors, year [References]**	**Study design**	**Study or protocol?**	**Single paper per study?**	**ASCOT measure (item)**	**Country**	**Sample description**	**Sample size**	**Summary of study purpose and key findings relevant to use of ASCOT**
St Clair Sullivan et al, 2023 [91]	RCT (feasibility)	Protocol	Yes	SCT4	UK	Older adults affected by frailty and living with HIV	84	A mixed methods feasibility RCT to evaluate comprehensive geriatric assessment for people living with HIV and frailty. Primary outcomes are recruitment and retention of participants, as well as completion of outcome measures by available participants. ASCOT-SCT4 will be collected as a secondary outcome, alongside EQ-5D and HIV PROM, at 6 and 12 months. Health service utilisation and satisfaction with care will also be assessed at each time point.
van Gameren et al, 2021 [65]	RCT	Protocol	Yes	SCT4	Netherlands	Older adults living at home, stratified by pre-frail and frail	256	A single-blinded multicentre RCT to evaluate an 'In Balance' intervention of education and physical exercise designed to reduce falls. Primary outcomes are number of falls and fall-related injuries over 12-month follow-up. Secondary outcomes include physical performance and activity measures, confidence, health status, quality of life (ASCOT-SCT4) and process evaluation and societal costs.
van Santen et al, 2020 [66]	RCT	Study	Yes	SCT4 (*Social*)	Netherlands	People with dementia (PwD) and their carers	112 dyads	A RCT to evaluate mixed physical and cognitive activity ('exergaming') at day care centres by comparison to care-as-usual. Primary outcomes were physical activity and mobility of the PwD. Secondary outcomes were physical, cognitive, emotional and social functioning, and quality of life for PwD. For carers, secondary outcomes were distress due to neuropsychiatric symptoms, burden, QoL, sense of competence and positive care experiences. The ASCOT-SCT4 *Social participation* item was collected as a secondary outcomes at baseline, 3- and 6-months for PwD. No significant effect was found for ASCOT-SCT4 *Social participation*.
Whitehead et al, 2024 [93]	RCT	Protocol	Yes	SCT4	UK	Older people with disabilities, living at home	272	Multicentre two-arm parallel group RCT ('BATH-Out 2') to evaluate expedited provision of level-access showers compared to usual-wait for older adults with disability, alongside a mixed methods process evaluation. RCT primary outcome is the older person's well-being (physical component of SF-36) at 4 weeks after the shower installation. Secondary outcomes include SF-36 mental component, self-report falls, health and social care resource use, ASCOT-SCT4 SCRQoL, fear of falling, ADLs (including bathing) and rating of perceived difficulty in bathing.
Whitehead et al, 2018 [94]	RCT (feasibility)	Study	Yes	SCT4	UK	Older people with disabilities, living at home	60	A feasibility RCT ('Bath-Out') to evaluate bathing adaptations for older adults. Outcomes were assessed at 3, 6 and 9 months, to include ASCOT-SCT4 SCRQoL among other outcomes (e.g. physical and mental status, ADLs and falls). The study found an improvement from baseline in both groups on all outcome measures (including ASCOT), following adaptation completion.
Whitehead et al, 2016 [92]	RCT (feasibility)	Study	Yes	SCT4	UK	Older people who were referred to a reablement service	30	A feasibility RCT ('OTHERS') to evaluate an occupational therapy intervention delivered within a homecare reablement service. Outcomes were collected at baseline and six-months, including ASCOT-SCT4. It was found that it was feasible to recruit participants, deliver the intervention and collect outcomes that were responsive to change, although the choice of primary outcome for a full trial was unclear.
Zhang et al, 2022 [61]	RCT	Protocol	Yes	SCT4	Australia	Older people living at home	195	A protocol for a single-blinded stratified RCT to evaluate the *Smarter, Safer Homes* (SSH) platform for self-monitoring and management for older people living at home, their family carers and care providers. Outcomes will be collected at baseline, 6- and 12-months, with ASCOT-SCT4 (collected using the Australian Community Outcomes Measurement (ACCOM) questionnaire) as the primary outcome.
Beresford et al, 2019 [96]	Mixed methods	Study	Yes	SCT4	UK	Older adults (50+ years) with dementia using reablement	T0: 184<br>T1: 128<br>T2: 59	A mixed-methods comparative evaluation of reablement service models investigating factors that have an impact on outcomes, costs and cost-effectiveness, and user and practitioner experiences. In WP2 (a study of three reablement services, each representing a different model of delivery), data were collected on health (EQ-5D-5L) and social care related (ASCOT-SCT4) quality of life, functioning, individual and service characteristics, and resource use. ASCOT-SCT4 was collected at: entry (T0), discharge (T1) and six months post-discharge (T2). There was a statistically significant increase in SCRQoL from T0 to T1, and no significant difference from T1 to T2. Comparisons between service models were not possible due to the smaller than expected sample sizes.

Authors, year [References]	Study design	Study or protocol?	Single paper per study?	ASCOT measure (item)	Country	Sample description	Sample size	Summary of study purpose and key findings relevant to use of ASCOT
Bertini et al, 2024 [101]	Mixed methods	Protocol	Yes	INT4	UK	Older adults resident in care homes	100	A protocol for a mixed methods longitudinal study of the impact of COVID outbreaks on care home residents' QoL, wellbeing, loneliness, functional ability and use of space. The study has two arms: (1) mixed methods interview using ASCOT-INT4 and (2) ethnographic observation. In (1), ASCOT-INT4 will be used to collect pre-post data at t0 and t1 (3-4 weeks later) for 100 residents, of whom half will be resident in a care home without COVID outbreak vs. residents in a home with COVID outbreak. Data will only be collected from residents with capacity to consent and participate in an interview, with data collected using ASCOT-INT4 (current only) alongside other structured data.
Burfeindt et al, 2024 [70]	Mixed methods	Protocol	Yes	SCT4	Germany	Older adults resident in care homes	TBC - all residents in 20 nursing homes	A protocol of an implementation and mixed methods evaluation study for a complex intervention to apply a standardised calculation to determine staffing levels ('Algorithm 1.0'). A pre- and post-test design (descriptive analysis) will be used to compare the effects of the intervention. Among other indicators, a paper-based survey of all residents in the 20 nursing homes will collect QoL using EQ-5D, ASCOT-SCT4 (self-report) and QUALIDEM (proxy report).
Clarke et al, 2018 [99]	Mixed methods	Study	Yes	SCT4	UK	People with dementia and their carers, staff and stakeholders	People with dementia (n=47), their carers (n=54) and staff and stakeholders (n=82)	A mixed methods study to explore the impact of active management of dementia on people's QoL and resilience. The study collected and reported ASCOT-SCT4 outcomes for people with dementia and their carers, both using ASCOT-SCT4, as well as from qualitative interviews. ASCOT-SCT4 data were collected at all study 40 sites, as well as at in-depth case study sites (n=8). ASCOT-SCT4 data were reported to map outcome-related needs to be addressed, which were notable across the sample for the ASCOT domains of *Control over daily life*, *Occupation* and *Accommodation comfort and cleanliness*.
Glasby et al, 2018 [97]	Mixed methods	Study	Yes	INT4 (*Control*, *Occupation*)	UK	Manager/senior staff of care providers<br>People using services/carers	32<br>106	A mixed methods study to compare the costs and outcomes of care delivery by micro-care providers (n=17) to small (n=4), medium (n=4) and large (n=2) providers of homecare, residential or day care services for older adults and/or adults with learning disabilities, across three areas in England. A survey used questions from the ASCOT-INT4 for two domains (*Control over daily life, Occupation*). The authors consider costs and outcomes, using ASCOT-INT4 data to show that micro-providers represent better value for money, but they acknowledge limitations due to small sample size and variability of the comparator group.
Gridley et al, 2019 [98]	Mixed methods	Study	Yes	Carer SCT4	UK	Unpaid carers of people with dementia	346 (158 intervention, 188 control)	A mixed methods evaluation of a specialist nursing intervention (Admiral Nurses (AN)) to support carers of people with dementia. One strand of the study involved data collection from 32 local authorities (16 with and 16 without the intervention). ASCOT-Carer SCT4 was selected as the outcome measure for 'impact on carers' QoL' based on a mapping exercise and ranking by carers (n=35) of a shortlist of six validated measures. Instrumental variable (IV) analysis for outcomes and costs, with travel time as an instrument, aligned with findings from regression analysis (no control for selection bias) and propensity score matching. None of the outcomes were significant for the intervention vs control, except ASCOT-Carer, which was weakly significant (10% level); carers using the AN intervention had better QoL outcomes than those who did not. The analysis found little difference in costs between AN and non-AN carers. As this study took place before preference weights were available for the ASCOT-Carer, the analysis was not able to calculate a measure akin to a QALY to explore cost-effectiveness.

Authors, year [References]	Study design	Study or protocol?	Single paper per study?	ASCOT measure (item)	Country	Sample description	Sample size	Summary of study purpose and key findings relevant to use of ASCOT
Orellana et al, 2020 [100]	Mixed methods	Study	Yes	INT4	UK	Older adults using traditional day centre services	23	A mixed methods case study design to determine the characteristics of older adults attending day centres, their reasons for attending and their outcomes. The study combined qualitative interviews with survey data collection of the participants' socio-demographics and their social network, health or wellbeing using validated tools (including ASCOT-INT4). There was considerable overlaps between the domains of ASCOT-INT4 and the 'outcome' themes identified in the qualitative interviews (6 of 8 ASCOT-INT4 domains aligned with 8/10 of the qualitative themes). Using the INT4 method, data collected from 22/23 participants showed the positive impact of day centre attendance on quality of life (average 'gain' in preference-weighted SCRQoL of 0.18). Statistically significant improvements in SCRQoL were observed in two ASCOT-INT4 domains (*Social participation*, *Occupation*). These findings were aligned to the findings from the qualitative data, collected from participants by interview.
Low et al, 2018 [63]	Mixed methods	Study	Yes	CH3	Australia	Older adults resident in care homes	Baseline (n=80) 12-month follow-up (n=69)	A pre-post mixed methods study to explore the feasibility of LifeFul, a relationship reablement-focussed culture change programme in residential care. Data were collected at baseline and follow-up at 12 months. Qualitative data was collected via focus groups and interviews with staff/managers to explore fidelity and adherence to the programme, as well as acceptability, enablers and barriers. Quantitative data were also recorded for staff recruitment/retention and resident-level outcomes, including ASCOT-CH3 SCRQoL. The study found improvement in the ASCOT-CH3 domains of *Personal safety, Occupation, Dignity* and overall ASCOT-CH3 SCRQoL between t0 and t1; however, the authors note the potential impact of ceiling effects on sensitivity.
Rand et al, 2020 [26]	Mixed methods	Study	No (IIASC)	Carer INT4	UK	Unpaid carers of people using community social care services	29 (qualitative interviews) 316 (survey)	A mixed methods study of carers of people using publicly funded social care in England, to explore whether and how formal social care services affect carers' SCRQoL. It combined insights from 29 qualitative interviews with carers (with ASCOT-Carer domains applied as a thematic framework) and quantitative data (ASCOT-Carer INT4) collected from a survey of 316 carers. The qualitative interviews provided insights into carers' experiences and views of how services have a positive, neutral or negative impact on their quality of life, across the seven ASCOT-Carer domains. No impact was reported when there was no underlying need (i.e. QoL is already ideal), mismatch between carers' needs and the social care intervention, when the care provided was insufficient in quality or quantity, or when carers did not have the time or space to engage with the services offered. Positive/negative effects were related to whether services alleviated or added to burden, helped carers to reappraise and manage their role, and/or alleviate or adds to subjective burden. Of the survey sample (n=316), 75.9% reported positive impact of services on QoL overall, with 21.9% reporting no impact. Only 2.2% reported an overall negative impact. As expected, SCRQoL 'gain' was related to cost-weighted intensity of services.
Ristolainen et al, 2022 [67]	Mixed methods	Protocol	Yes	INT4	Finland	Older adults using homecare	200 (100 intervention, 100 control)	A mixed methods study to determine the cost effectiveness and process of day activity services for older adults in Finland. The intervention group will access a day activity service in addition to homecare, with the control group continuing with homecare only. Quantitative data will be collected at baseline, 3 and 6 months. The primary outcome is social inclusion, with loneliness and SCRQoL (ASCOT-INT4) as secondary outcomes. ASCOT-INT4 will be collected at 3 and 6 month follow-up for the n=100 older adults in the intervention group only. ASCOT-INT4 is not proposed to be used in the qualitative data collection for the study.
Siette et al, 2021 [58]	Mixed methods	Study	Yes	SCT4	Australia	Older adults living at home	56	A mixed methods study to evaluate an excursion programme for older adults in Australia. The study had two stages: (1) a pre and post-test survey of older adults who received the service for six months and (2) semi structured interviews with care staff, older people and their carers (n=24). ASCOT-SCT4 was used in the first phase only. ASCOT-SCT4 data collection was embedded in the service provider's routine data collection for assessment and feedback, by self-complete survey. Of the 110 responses at baseline, there were only 56 who also completed at six month follow-up. Pre and post test analysis of these cases (n=56) found a significant improvement at follow-up. Those who did not complete the follow-up had significantly higher SCRQoL at baseline than those who did.

Authors, year [References]	Study design	Study or protocol?	Single paper per study?	ASCOT measure (item)	Country	Sample description	Sample size	Summary of study purpose and key findings relevant to use of ASCOT
Smith et al, 2018 [73]	Mixed methods	Study	Yes	CH3	UK	Older adults resident in care homes	72	A mixed methods study using ASCOT-CH3 to collect quantitative and qualitative (observational, interview, focus group) data on residents' SCRQoL to explore the concept of meaningful activity or being occupied among care home residents. Low levels of being occupied among care home residents are discussed in light of evidence of the impacts of staffing levels (i.e. unavailability of staff to support occupation) and working practices (i.e. prioritising basic health and care needs over higher-order QoL domains, like *Occupation*).
Towers et al, 2023 [76]	Mixed methods	Protocol	Yes	Proxy	UK	Older adults resident in care homes	960	A protocol for a mixed methods longitudinal study to pilot a minimum dataset for older adult care homes. The study will pilot the collection of resident-level QoL using standardised measures (EQ-5D-5L Proxy, QUALIDEM, ICECAP-O (by proxy) and ASCOT-Proxy) rated by care staff proxy report as part of routine care. Proxy report by care staff was chosen as the method of data collection given prior UK evidence of the high % missing data from self-report or family proxy report, which reflects the profile of the UK care home population. A single item QoL overall rating was also included for self-report, if feasible, either alone or with staff support.
Wistow et al, 2016 [79]	Mixed Methods	Study	Yes	INT4 by proxy*<br><br>**Informal adaptation*	UK	Adults with intellectual disability using Circles of Support	5	An exploratory mixed methods study to evaluate *Circles of Support*, a care planning approach that engages the person, their family, and other supporters to help the person make purposeful and meaningful life choices. ASCOT-INT4 was informally adapted by the research team to (1) collect information from care staff or family proxies and (2) to apply the INT4 method, without the 'filter' questions. The ASCOT results showed that the Circle had impacted positively on SCRQoL (ASCOT-INT4 average score: 0.82 compared to an expected score of 0.21 without the *Circle* = ASCOT SCRQoL gain of 0.61).
Bauer et al, 2017 [102]	Cost consequence analysis	Study	Yes	SCT4	UK	Older adults (55+ years) using a help-at-home scheme	24	A cost consequence study of a voluntary sector provided help-at-home scheme in England. Data were collected using ASCOT-SCT4 at both baseline and follow-up (5-6 months) for 24 (of 41) older adults. Costs were calculated from local government, central government and individual perspectives. Pre/post comparison using T-tests found a non-significant increase in SCRQoL between baseline and follow-up, across all ASCOT-SCT4 domains.
Damant et al, 2017 [104]	Scoping literature review	Study	Yes	SCT4	UK	Older people living at home	N/A	A scoping literature review of the effects of digital engagement (use of mobile phones, the internet and computers) on older people's QoL. The authors applied framework analysis to the identified papers. They applied the QoL domains from ASCOT-SCT4 and WHOQoL to create a theoretical framework of six QoL domains to analyse and interpret the QoL impacts for older adults. This framework included the ASCOT-SCT4 domains *of Control over daily life, Personal safety, Social participation* and *Occupation*, as well as reference to *Food and Drink, Personal and Accommodation comfort and cleanliness* and *Dignity*.
Golding-Day et al, 2020 [105]	Qualitative	Study	Yes	SCT4	UK	Older people, living at home,<br>and their carers	13<br><br>3	An extended follow-up qualitative study of older adults, who had a bathing adaptation to install an accessible shower into their home, to understand their experiences and outcomes. This study builds on a nested qualitative interview study completed as part of a feasibility RCT (BATH-OUT, see Whitehead et al). The study also reports quantitative data collected at baseline and follow-up (3, 6 and 9 months) using: SF-36, EQ-5D-5L, ASCOT-SCT4, Barthel Index, perceived ease and independence in bathing, and short falls efficacy scale. ASCOT-SCT4 scores were found to improve from baseline for overall SCRQoL and across ASCOT-SCT4 domains. These quantitative findings were compared to the qualitative findings, which aligned to the change in scores for *Personal cleanliness*, *Control over daily life, Personal safety, Occupation* (doing things I value and enjoy) and *Social participation*.
Rand & Malley, 2014 [22]	Qualitative	Study	No (IIASC)	Carer INT4	UK	Carers of adults using community-based social care	31	A qualitative study of carers' QoL and experiences of caring, using data collected from cognitive interviews with the ASCOT-Care INT4. Three themes were identified: services 'for' carer and 'for' the people they support; carers access to services; and carers as 'expert partners in care'. Of these themes, the first theme drew on the ASCOT-Care's concept of SCRQoL, with exploration of the ways in which services can directly or indirectly affect carers' SCRQoL and the tensions or trade-offs in SCRQoL that affect carers in their caring role.

Authors, year [References]	Study design	Study or protocol?	Single paper per study?	ASCOT measure (item)	Country	Sample description	Sample size	Summary of study purpose and key findings relevant to use of ASCOT
Robertson et al, 2024 [62]	Pre-post pilot study	Study	Yes	Carer SCT4	Australia	Carers of children with developmental and epileptic encephalopathy	72	As part of a larger pre-post pilot study to evaluate an information linker service, the relationship between ASCOT-Carer SCRQoL and health literacy, illness perceptions and carer activation were explored. It was found that ASCOT-Carer SCRQoL was significantly associated with cognitive and emotional representations of the illness, but not coherence - nor also, health literacy or carer activation. The SCRQoL scores were also reported descriptively to highlight high levels of unmet need among carers, especially for *Occupation* and *Self-care*.
Towers et al, 2016 [75]	Implementation	Study	Yes	CH3	UK	Older adults resident in care homes	58	An implementation study of a QoL feedback intervention that used the ASCOT-CH3 in four older adult care homes. It was found that expected ASCOT-CH3 SCRQoL (without services, as an indicator of underlying need) declined from baseline before the intervention (T1) to follow-up (T2). However, the reduction in current QoL was not statistically significant, which implies that services were able to respond to increasing need over time. Qualitative evidence from feedback sessions with staff indicated that the intervention was acceptable and had led to reflection and implementation of changes by managers in response to feedback.
Woolham et al, 2017 [103]	Retrospective comparative	Study	Yes	SCT4	UK	Older people (75+) using direct payments or managed personal budgets	339	A retrospective comparative study to determine whether direct payments improve outcomes for older people accessing social care. Using data collected in a postal survey of people using social care services across three English local authorities, no significant difference in outcomes was found between older adults using direct payments compared to managed personal budgets for health-related QoL (EQ-5D), care-related QoL (ASCOT-SCT4 SCRQoL) or stress (Sheldon perceived stress Scale).
